# Editor’s choice 2019: Oxidative stress and antineoplastic agents

**DOI:** 10.17179/excli2020-3284

**Published:** 2020-12-15

**Authors:** Ahmed Ghallab

**Affiliations:** 1 Forensic Medicine and Toxicology Department, Faculty of Veterinary Medicine, South Valley University, Qena, Egypt

## ⁯

Each issue of EXCLI Journal contains recommendations of ten articles of the previous year chosen by the editorial board. Articles chosen from the 2019 issue include a wide range of topics with a focus on oxidative stress and antineoplastic agents. 

*• Induction of apoptosis by piperine in human cervical adenocarcinoma via ROS mediated mitochondrial pathway and caspase-3 activation*

Piperine (1-piperoylpiperidine) concentration dependently induces apoptosis in the human cancer cell line HeLa (Jafri et al., 2019[4]). The compound was shown to cause an increase in reactive oxygen species and a G2/M phase arrest. It will be interesting to learn more about the anticancer potential of this compound *in vivo. *

*• Hesperidin ameliorates bleomycin-induced experimental pulmonary fibrosis via inhibition of TGF-beta1/Smad3/AMPK and IkappaBalpha/NF-kappaB pathways*

One of the most severe side effects of the antineoplastic compound bleomycin is pulmonary fibrosis. Interestingly, the flavonoid hesperidin ameliorated bleomycin induced lung fibrosis in rats (Zhou et al., 2019[10]). However, it remains to be analyzed if co-administration of hesperidin together with bleomycin also reduces the antineoplastic effect of bleomycin.

*• Antiviral activity of flavonoids present in aerial parts of Marcetia taxifolia against Hepatitis B virus, Poliovirus, and Herpes Simplex virus in vitro*

The authors studied the antiviral effects of compounds isolated from the aerial parts of the neo-tropical plant *Marcetia taxifolia* (Ortega et al., 2019[6]). Methoxyflavones were identified as possible lead compounds with a broad antiviral activity. 

*• Auraptene-induced cytotoxicity mechanisms in human malignant glioblastoma (U87) cells: role of reactive oxygen species (ROS)*

The prenyloxy coumarin Auraptene was shown to be cytotoxic in human glioblastoma cells and to upregulate p21, while cyclin D_1_ was suppressed (Afshari et al., 2019[1]). Therefore, the antineoplastic capacity of this compound should be studied in mouse tumor models. 

*• Up-regulation of long non-coding RNA-PCAT-1 promotes invasion and metastasis in esophageal squamous cell carcinoma ‎*

The authors demonstrated that the long non-coding RNA prostate cancer associated transcript-1 ncRNA (lncRNA-PCAT-1) is up-regulated in 150 esophageal squamous carcinomas compared to adjacent non-cancer tissue (Razavi and Ghorbian, 2019[7]). 

*• Efficacy and tolerability of fourteen-day sequential quadruple regimen: pantoprazole, bismuth, amoxicillin, metronidazole and or furazolidone as first-line therapy for eradication of Helicobacter pylori: a randomized, double-blind clinical trial‎*

This is a randomized, double-blind clinical trial conducted with 344 patients to study efficacy and safety of bismuth-based quadruple therapy compared to a modified regimen to eradicate *Helicobacter pylori* (Mansour-Ghanaei et al., 2019[5]). Efficacy was highest for the bismuth-based quadruple therapy. 

*• Immunoregulatory, proliferative and anti-oxidant effects of nanocurcuminoids on adipose-derived mesenchymal stem cells*

The influence of nano-formulations of curcuminoids on mesenchymal stem cells was analyzed (Yousefi et al., 2019[9]). Low concentrations reduced inflammatory cytokines and showed anti-apoptotic effects. However, at higher concentrations the expression of TGFbeta was increased. 

*• Rhomboid antigens are promising targets in the vaccine development against Toxoplasma gondii*

Approximately 30 % of humans are seropositive for *Toxoplasma gondii*. The authors reviewed the recent progress in the development of vaccines based on rhomboid proteases, a class of serine proteases that play a role during the invasion of the parasites (Foroutan et al., 2019[3]). 

*• Cell phone addiction and psychological and physiological health in adolescents*

The author reviews the influence of the excessive use of cell phones on the psychological health of adolescents (Shoukat, 2019[8]). 

*• Exposure to effluent from pharmaceutical industry induced cytogenotoxicity, hematological and histopathological alterations in Clarias gariepinus (Burchell, 1822)*

Effluents from pharmaceutical companies may contain toxic xenobiotics. The authors showed significant genotoxic effects of pharmaceutical effluents, which may lead to adverse effects on aquatic biota (Alimba et al., 2019[2]).

## Conflict of interest

The author declares no conflict of interest.
